# Immunostimulatory effects of marine algae extracts on in vitro antigen‐presenting cell activation and in vivo immune cell recruitment

**DOI:** 10.1002/fsn3.3605

**Published:** 2023-08-21

**Authors:** Thi Len Ho, Jueun Lee, So Yeon Ahn, Dong‐Ha Lee, Woo‐Jin Song, Inhae Kang, Eun‐Ju Ko

**Affiliations:** ^1^ Interdisciplinary Graduate Program in Advanced Convergence Technology & Science Jeju National University Jeju Republic of Korea; ^2^ Department of Veterinary Medicine, College of Veterinary Medicine Jeju National University Jeju Republic of Korea; ^3^ Veterinary Medical Research Institute, Jeju National University Jeju Republic of Korea; ^4^ Department of Food Science and Nutrition Jeju National University Jeju Republic of Korea

**Keywords:** DCs, immunostimulatory effects, macrophages, marine algae extracts

## Abstract

Marine algae are photosynthetic eukaryotic organisms that are widely used as sources of food, cosmetics, and drugs. However, their biological and immunological effects on immune cells have not been fully elucidated. To unravel their immunological activity and broaden their application, we generated antigen‐presenting cells (APCs), including dendritic cells (DCs) and macrophages, from mouse bone marrow cells and treated them with six different marine algae extracts (MAEs). We evaluated cell viability, activation marker expression, and pro‐inflammatory cytokine production by APCs after 2 days of MAE treatment. All six MAEs significantly induced cytokine production of APCs, among which *Pyropia yezoensis* (PY), *Peyssonnelia caulifera* (PC), and *Meristotheca papulosa* (MP) extracts exhibited the strongest effect. *Cladophora wrightiana var. minor* (CW) extract moderately upregulated cytokine levels but increased the expression of activation markers on DCs. Moreover, PY, PC, MP, *Sargassum pectinifera* (SP), and *Caulerpa okamurae* (CO) pre‐treated APCs effectively stimulated T‐cell proliferation and cytokine production. Furthermore, the mice injected with MAEs exhibited higher cytokine (TNF‐α, IL‐6, and IL‐1β) production as well as enhanced innate immune cell recruitment capacities (DCs, monocytes, neutrophils, and natural killer cells) in the peritoneal cavity of the mice compared to those of the non‐treated mice. Therefore, all MAEs exhibited immunostimulatory potential, with PY, PC, CW, and MP extracts being the most effective in stimulating immune responses and cell activation. To the best of our knowledge, this is the first study to determine the immunomodulatory activities of six MAEs both in vitro and in vivo.

## Graphical abstract

Six different marine algae extracts (MAEs) activated bone marrow‐derived dendritic cells and bone marrow‐derived macrophages by upregulating activation marker expressions and producing cytokines. In addition, the mice injected with MAEs exhibited higher cytokine production as well as enhanced innate immune cell recruitment capacities. This study can unravel the immunological activity of MAEs and broaden their applications in food and pharmaceutical fields.

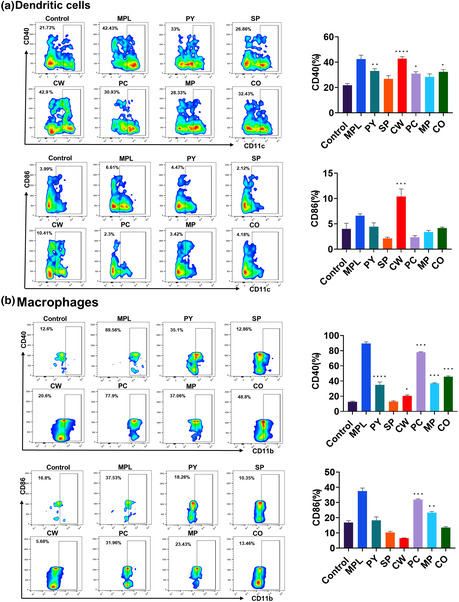

## 1 INTRODUCTION

The innate immune system plays a critical role in providing the host with an initial defense against infection (Iwasaki & Medzhitov, 2010). Antigen‐presenting cells (APCs) such as dendritic cells (DCs) and macrophages can stimulate innate immunity and bridge innate and adaptive immunity through T‐cell activation (Gaudino & Kumar, 2019; Mellman & Steinman, 2001). Both DCs and macrophages respond to various antigens by sensing and capturing pathogens via pattern recognition receptors, such as toll‐like receptors (Chaperot et al., 2000; Grabowska et al., 2018; Mosaheb et al., 2017). After capturing antigens, APCs process the antigens and present the antigenic peptide on their major histocompatibility (MHC) class II molecules to initiate antigen‐specific T‐cell responses. To promote interactions with T‐cells, APCs upregulate their surface molecules, such as CD40 and CD86, as well as pro‐inflammatory cytokine production, such as tumor necrosis factor (TNF)‐α, interleukin (IL)‐6, and IL‐12 (de Saint‐Vis et al., 1998). These two types of APCs, namely macrophages and DCs, have been used to investigate the biological and immunological effects of many new compounds, such as drugs and extracts, to discover their function and application in immune therapy (DeNardo & Ruffell, 2019; Gardner et al., 2020; Sadeghzadeh et al., 2020; Xia et al., 2020).

Recently, algae have attracted the attention of scientists as a potential source for novel therapeutic compounds. Marine algae are extremely diverse organisms that live mainly in the sea (Veluchamy & Palaniswamy, 2020) and have been used in many industries, such as food (Ngo et al., 2011), cosmetics (Alves et al., 2020), and pharmaceuticals (Barzkar et al., 2021; Molinski et al., 2009). Marine algae extracts (MAEs) contain vital components that treat various diseases, such as tumors (Vishchuk et al., 2013), obesity (Robbens et al., 2008), and infectious diseases (Wang, Wu, et al., 2017). This could be due to the immunomodulatory and anti‐inflammatory activities of MAEs (Bhardwaj et al., 2021; Riccio & Lauritano, 2019). Moreover, some marine algae exhibit strong antiviral activity both in vitro and in vivo (Nagarajan & Mathaiyan, 2015), which was recently validated against severe acute respiratory syndrome coronavirus 2 (SARS‐CoV‐2) (Andrew & Jayaraman, 2021; Hans et al., 2021). Thus, MAEs can also be used for the development of vaccine adjuvants (Sanina, 2019; Woods et al., 2017).

Each marine algae has its own unique biochemical composition. According to an extensive body of literature, marine algae contain abundant bioactive metabolites, such as polyphenols, sulfated polysaccharides, sterols, amino acids, and accessory pigments, in addition to dietary fiber, minerals, lipids, proteins, omega‐3 fatty acids, and vitamins. The extracts derived from various algal species display different ratios of primary constituents: phenolic compounds and flavonoids. Particularly, extracts from green and brown algae are often found to contain a greater proportion of phenolic compounds compared to red algae extracts. More than 30,000 species of marine algae exist, but only a few of them have been used (Guiry, 2012; Leandro et al., 2019; Lee et al., 2021; Matanjun et al., 2008; Menaa et al., 2021; Pereira, 2022; Septiyanti et al., 2019; Wang, Li et al., 2017).

To broaden the application of marine algae in biomedical and pharmaceutical fields, it is necessary to investigate their immunomodulatory effects and the underlying mechanisms. Therefore, in this study, we aimed to preliminarily screen the immunostimulatory effects of extracts from six marine algae species, which are widely distributed in the coastal areas of Korea (*Pyropia yezoensis*, PY, red algae; *Sargassum pectinifera*, SP, brown algae; *Cladophora wrightiana var. minor*, CW, green algae; *Peyssonnelia caulifera*, PC, red algae; *Meristotheca papulose*, MP, red algae; and *Caulerpa okamurae*, CO, green algae). We investigated the effect of the MAEs on DCs and macrophages, activation of marker expression, cytokine production, and T‐cell proliferation, as well as cytotoxicity in an in vitro system. Moreover, we evaluated in vivo cell recruitment and cytokine/chemokine production in mice after intraperitoneal injection of the MAEs.

## 2 MATERIALS AND METHODS

### 2.1 Animals and reagents

For this study, we purchased 6–7‐week‐old female BALB/c and C57BL/6 mice from Orient Bio Co. The Institutional Animal Care and Use Committee of Jeju National University (Jeju, Korea) approved the protocols for the animal experiments (protocol number: 2021–0051). Monophosphoryl lipid A (MPL), the immunostimulatory positive control used in all experiments, was purchased from InvivoGen. All MAEs were prepared and provided by the Marine Bio Bank of Marine Biodiversity Institute of Korea (Chungcheongnam‐do, Korea, http://www.mabik.re.kr).

### 2.2 DC and macrophage generation and treatment

Bone marrow‐derived dendritic cells (BMDCs) and macrophages (BMDMs) were prepared as previously described (Kim et al., 2021; Ko et al., 2017). Initially, bone marrow cells were harvested from the femurs and tibias of the BALB/c mice. To generate BMDCs and BMDMs, the bone marrow cells were cultured in Roswell Park Memorial Institute 1640 medium containing 10% fetal bovine serum, 1× antibiotic‐antimycotic (Gibco), 20 ng/mL mouse granulocyte‐macrophage colony‐stimulating factor (mGM‐CSF), and 20 ng/mL mouse macrophage colony‐stimulating factor (mM‐CSF). Every 2 days for 6 days, the medium was replaced with fresh culture medium containing mGM‐CSF or mM‐CSF, and then the immature DCs (iDCs) and macrophages (iM) were harvested.

To investigate cell viability and cytokine production, 1 × 10^6^ cell/mL of iDCs and 5 × 10^5^ cell/mL of iM were seeded in 96‐well plates (200 μL/well) and treated with 0.1 μg/mL MPL or 20 μg/mL PY, SP, CW, PC, MP, or CO and then incubated at 37°C. After 2 days, the viability rate of the cells was evaluated using the 3‐(4,5‐dimethylthiazol‐2‐yl)‐2,5‐diphenyltetrazolium bromide (MTT) assay, whereas the levels of IL‐6, TNF‐α, and IL‐12p40 in the culture supernatant were determined using enzyme‐linked immunoassay (ELISA) kits (Invitrogen).

For evaluating the effects of MAEs on the activation marker expression on DCs and macrophages, 1 × 10^6^ cell/mL iDCs and 5 × 10^5^ cell/mL of iM were seeded in 6‐well plates (2.5 mL/well) and incubated in the presence of 0.1 μg/mL of MPL or 20 μg/mL of each MAE. After 2 days, the cells were harvested and stained with fluorescence‐labeled antibodies to determine cell phenotypes and evaluate the levels of activation marker expression.

### 2.3 Allogeneic mixed lymphocyte reaction (MLR)

To evaluate the lymphocyte‐stimulating effects of MAE‐treated DCs and macrophages, BMDCs and BMDMs were seeded and treated with MAEs for 2 days, and the conditions were similar to those of the activation marker expression experiment. Allogenic naïve lymphocytes were isolated from spleen cells of C57BL/6 mice as previously described (Lim et al., 2016). After staining with carboxyfluorescein succinimidyl ester (CFSE), the CFSE‐labeled lymphocytes (2 × 10^6^ cell/mL) were co‐cultured with MAE‐pre‐treated DCs (2 × 10^5^ cell/mL) and macrophages (1 × 10^5^ cell/mL) in a 96‐well U‐bottomed plate. After 5 days of incubation, the cells were harvested to identify T‐cell proliferation using flow cytometry, and the cell supernatant was used for the analysis of cytokine production using ELISA.

### 2.4 In vivo intraperitoneal injection

We performed intraperitoneal injection assay to evaluate cell recruitment in the peritoneal cavity of mice following MAE treatment. Initially, BALB/c mice (*n* = 6) were injected intraperitoneally with 200 μL of PBS containing MPL (1 μg/mouse) or MAEs (100 μg/mouse). After 6 and 24 h of injection (*n* = 3, each time point), the peritoneal cells and exudates were harvested by peritoneal lavage with 2 mL of PBS and then centrifuged at 1600 rounds per minute at 4°C for 5 min to isolate peritoneal cells. The cells were stained and analyzed using flow cytometry, and the levels of cytokines and chemokines in the peritoneal exudates were measured using ELISA.

### 2.5 Flow cytometry

DC and macrophage activation statuses were examined using flow cytometry. Before adding the antibody cocktail, the Fc receptors of DCs and macrophages were blocked. DCs were stained with CD11c‐PE/Cy7 (clone N418, eBioscience™), MHC class II (MHCII)‐PE (clone M5/114.15.2, eBioscience™), CD40‐BV605 (clone 3/23, BD OptiBuild™), and CD86‐FITC (clone GL1, BD Pharmingen™) antibodies to investigate the expression of activation markers. To activate the macrophages, CD11b‐APC (clone M1/70, BD Pharmingen™), MHC II (clone M5/114.15.2, eBioscience™), CD40‐BV605 (clone 3/23; BD Biosciences), and CD86‐FITC (clone GL1; BD Pharmingen™) antibodies were used. To evaluate T‐cell proliferation after co‐culture, co‐cultured lymphocytes were stained with CD3‐BV421 (clone 17A2; BD Horizon™), CD4‐PE/Cy7 (clone RM4.5, BD Pharmingen™), and CD8‐APC (clone 53–6.7, BD Pharmingen™). The phenotypes of the recruited cells in the peritoneal cavity after MAE treatment were determined by staining the peritoneal cells with a fluorescence‐labeled antibody cocktail after blocking the Fc receptor with a purified anti‐CD16/32 antibody. The antibodies used for this experiment were as follows: CD45‐PerCP (clone 30‐F11, BD Pharmingen™), anti‐mouse F4/80‐FITC (clone BM8, eBioscience™), MHC II (clone M5/114.15.2, eBioscience™), CD11c ‐PE/Cy7 (clone N418, eBioscience™), CD11b‐APC (clone M1/70, BD Pharmingen™), anti‐mouse CD206 (clone C068C2), and Ly6c‐AF700 (clone AL‐21, BD Pharmingen™). Flow cytometry was performed on a BD LSRII Fortessa and FACS Diva. The data were analyzed using FlowJo software (Version 10.1, Tree Star Inc.).

### 2.6 ELISA

To measure the cytokine and chemokine levels in the culture supernatant and peritoneal lavage fluid, IL‐6, TNF‐α, and IL‐12p40 ELISA kits (Invitrogen) and interferon‐gamma IFN‐γ, granzyme B, CCL2, and CCL5 ELISA kits (R&D Systems) were used as per the manufacturer's instructions.

### 2.7 Statistical analysis

All data are shown as the mean ± standard error, and one‐way ANOVA and Tukey's multiple comparison tests were performed for statistical analysis. All results were analyzed using GraphPad Prism software 9.2.0 (GraphPad Software Inc.). *p* < .05 was set as statistical significance.

## 3 RESULTS

### 3.1 Evaluation of cytotoxicity of MAEs in BMDCs and BMDMs

**FIGURE 1:**
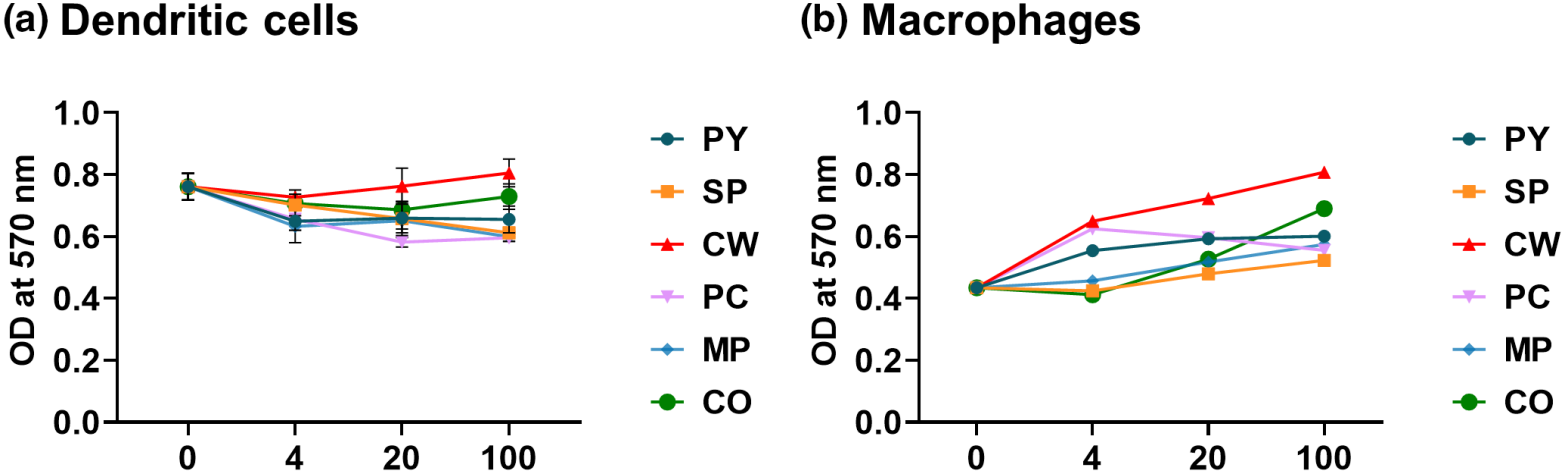
Cell viability after treatment with marine algae extracts. Bone marrow‐derived dendritic cells (a) and macrophages (b) were treated with different concentrations of each marine algae extract (marine algae extract; 4, 20, and 100 μg/mL) for 2 days, and MTT assay was performed. Data are shown as mean ± standard error.

To investigate whether MAE treatment affect cell viability or not, various concentrations (0–100 μg/mL) of each MAE were treated to BMDCs and BMDM for 2 days and MTT assay was performed (Figure 1). We observed that the viability of BMDCs slightly decreased after treatment with all MAEs (4 μg/mL). However, the viability of BMDCs did not decrease at higher concentrations (20 and 100 μg/mL), except in case of SP and PC treatments. SP treatment gradually decreased cell viability, and PC treatment exhibited the lowest cell viability at 20 μg/mL. In contrast, BMDMs exhibited increased cell viability in a concentration‐dependent manner with all MAE treatments, except PC. PC treatment exhibited the highest cell viability at 4 μg/mL; although the viability gradually decreased at higher concentrations of PC extract, but it was still higher than that of non‐treated BMDMs (Figure 1). For further experiments, the concentration at 20 μg/mL was used for all MAEs.

### 3.2 MAE treatment enhanced pro‐inflammatory cytokine production from DCs and macrophages

**FIGURE 2:**
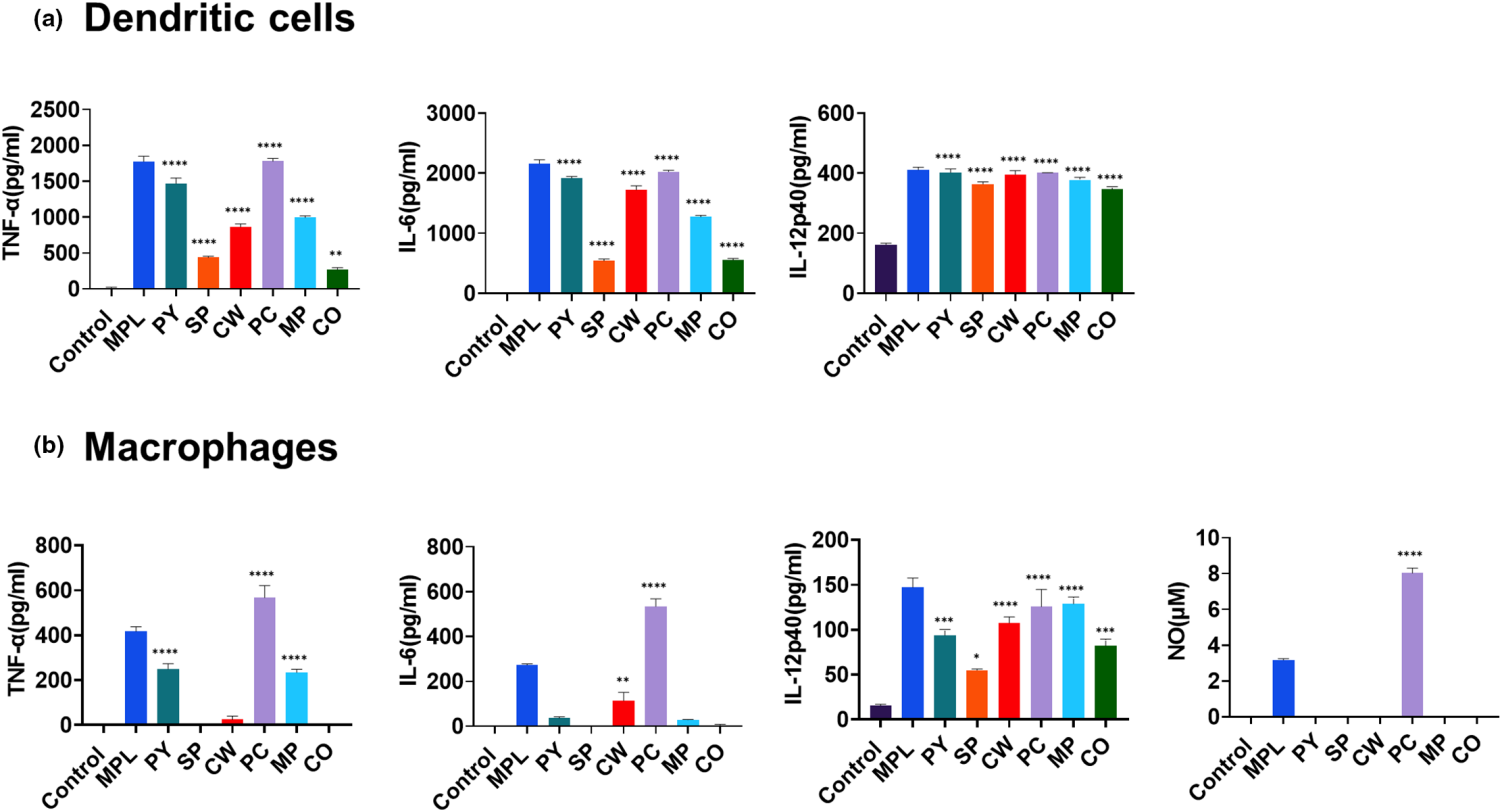
Cytokine production of marine algae extract (MAE)‐treated bone marrow‐derived dendritic cells (BMDCs) and bone marrow‐derived macrophages (BMDMs). BMDCs (a) and BMDMs (b) were treated in vitro with MAEs or monophosphoryl lipid A (MPL) (positive control, 0.1 μg/mL MPL; 20 μg/mL each MAE). After 2 days, the level of nitric oxide (NO), interleukin (IL)‐6, tumor necrosis factor (TNF)‐alpha, and IL‐12p40 was determined by ELISA. Data are shown as mean ± standard error. One‐way ANOVA and Tukey's multiple comparison tests were performed for statistical analysis. *p < .0332; **p < .0021; ***p < .0002; and ****p < .0001 compared to control group.

To evaluate the effects of MAEs on the cytokine production of BMDCs and BMDMs, 20 μg/mL of MAEs were treated with immature BMDCs and BMDMs. After two‐day culture, the levels of cytokines in the cell culture supernatants were measured by ELISA. MPL (0.1 μg/mL) was used as a positive control. We observed that MAEs significantly enhanced TNF‐α, IL‐6, and IL‐12p40 production of DCs (Figure 2a). In particular, when compared with control, PY, CW, PC, and MP treatments induced higher levels of TNF‐α and IL‐6, whereas SP and CO extracts induced lower levels of TNF‐α and IL‐6. However, all MAEs strongly induced IL‐12p40 production of DCs, which was similar to that of the control (MPL). In BMDMs, only PC treatment induced cytokine (TNF‐α, IL‐6, and IL‐12p40) production, as well as NO (Nitric Oxide) production (Figure 2b). In macrophages, PY and MP treatments induced TNF‐α secretion, and CW‐stimulated macrophages produced IL‐6. Similar to that in BMDCs, IL‐12p40 production was induced by all MAEs. Overall, MAEs enhanced pro‐inflammatory cytokine production in APCs, and PC exhibited the strongest stimulatory effects on both DCs and macrophages.

### 3.3 MAEs promoted the activation marker expression on BMDCs and BMDMs

**FIGURE 3:**
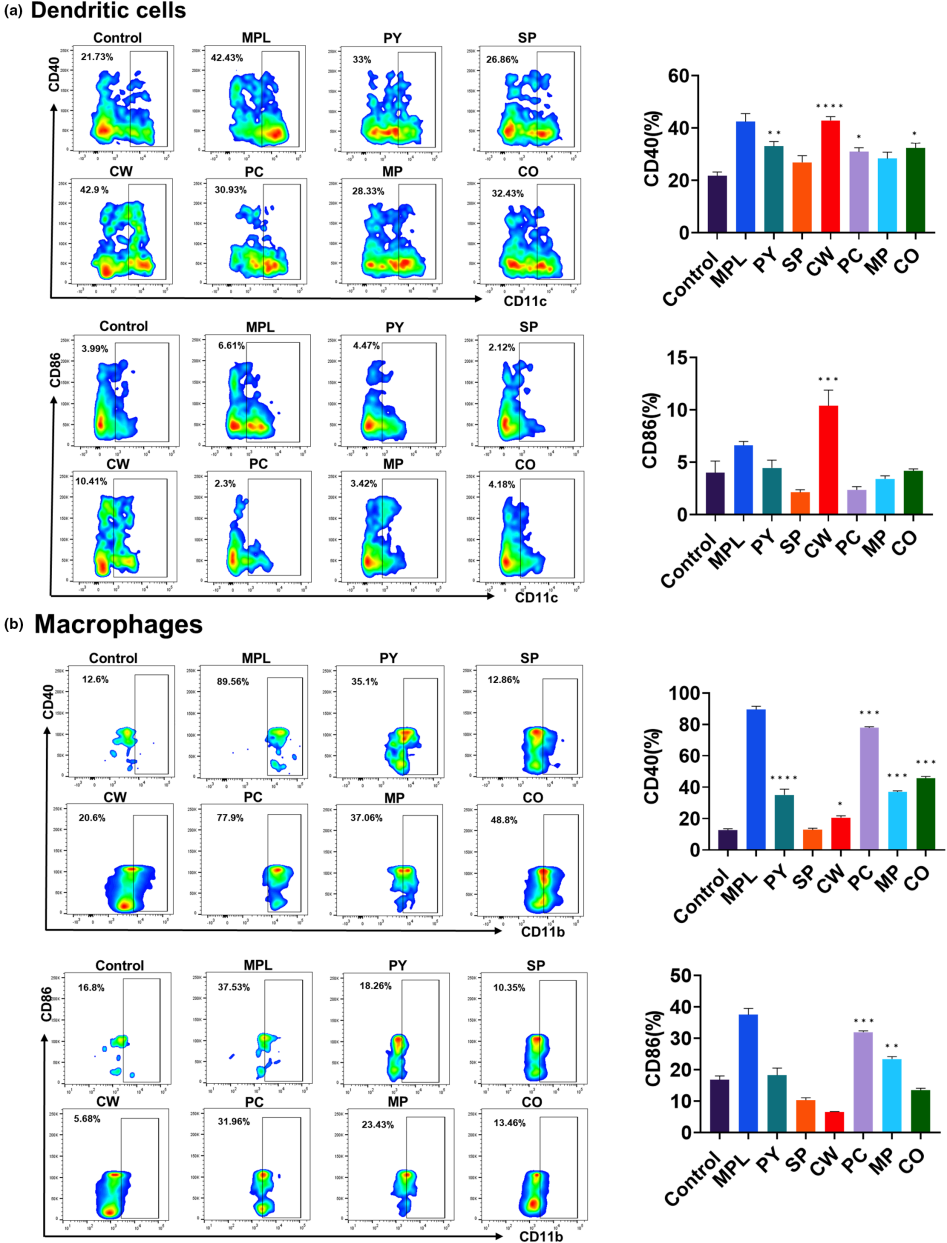
Expression of activation markers by bone marrow‐derived dendritic cells (BMDCs) and bone marrow‐derived macrophages (BMDMs) induced by marine algae extracts (MAEs). BMDCs and BMDMs were treated with MAEs or monophosphoryl lipid A (MPL) (positive control) in vitro (0.1 μg/mL MPL or 20 μg/mL each MAE). After 2 days, MHC class II, CD40, and CD86 expression on dendritic cells (DCs) (a) and macrophages (b) were determined by flow cytometry and analyzed by FlowJo. DCs and macrophages were gated by CD11c and CD11b surface markers, respectively. The representative data obtained from the flow cytometry analysis were shown in pseudo‐color smooth plots. Data are shown as mean ± standard error. One‐way ANOVA and Tukey's multiple comparison tests were performed for statistical analysis. *p < .0332; **p < .0021; ***p < .0002; and ****p < .0001 compared to control group.

Next, we examined whether MAE treatment enhanced the activation marker expression of BMDCs and BMDMs. After 2 days of MAEs treatment, the expression of activation markers on DCs and macrophages was determined by flow cytometry. CD40 expression on DCs was significantly increased by PY, CW, PC, and CO treatments, especially by CW treatment, whereas CD86 expression was enhanced only on CW‐treated DCs (Figure 3a). However, on macrophages, CD40 expression was enhanced by PY, CW, PC, MP, and CO treatments, whereas CD86 expression was induced only by PC and MP treatments (Figure 3b). CW treatment did not enhance the expression of activation markers on macrophages to the same extent as that on DCs. Thus, CW treatment exhibited strong stimulatory effects on activation marker expression of DCs but not of macrophages. PC and MP treatments induced both CD40 and CD86 expressions on macrophages.

### 3.4 DCs and macrophages activated by MAEs induced T‐cell proliferation and IFN‐γ production after in vitro co‐culture

**FIGURE 4:**
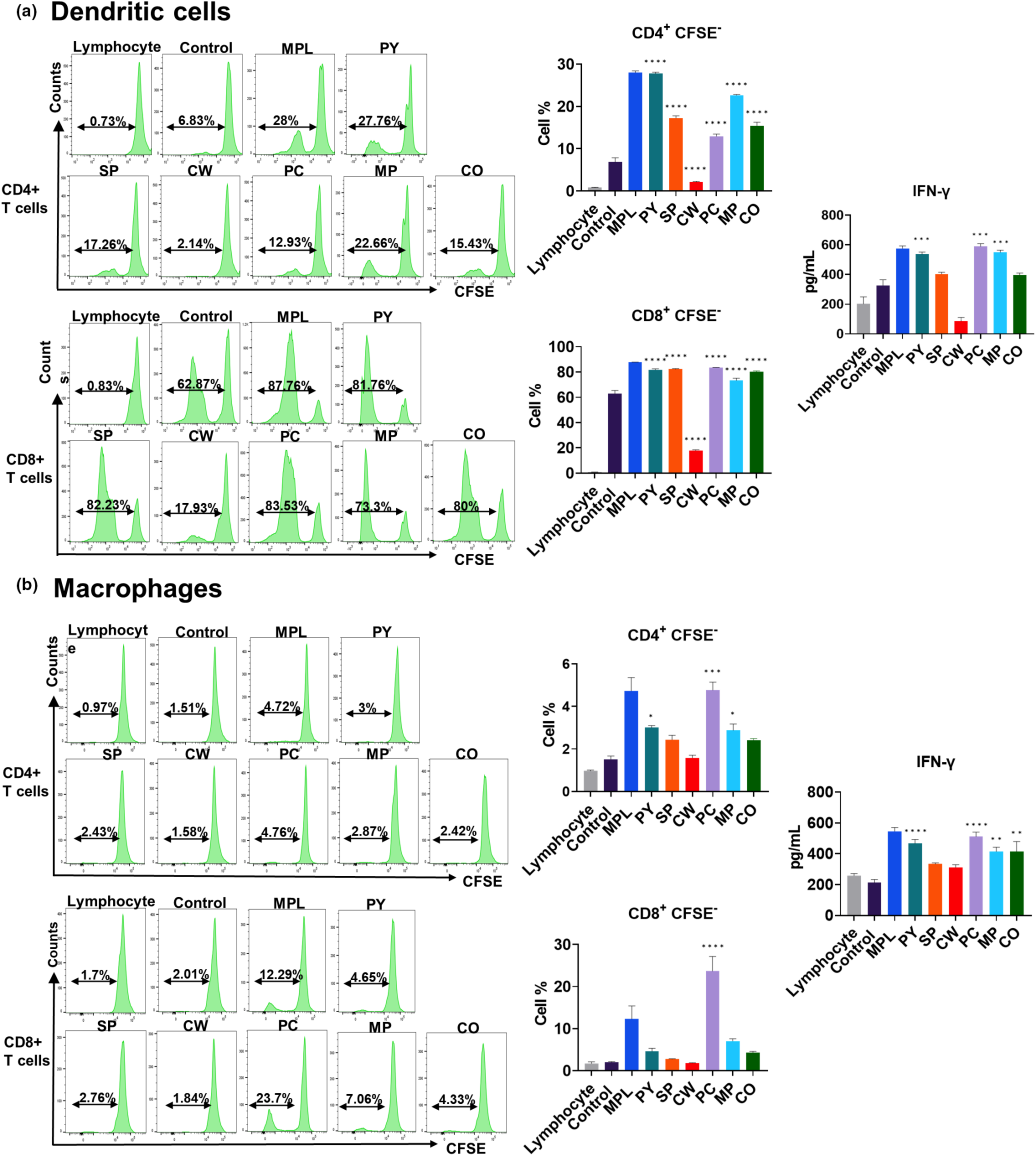
T‐cell proliferation and interferon gamma (IFN‐γ) production by T‐cells after co‐culture with marine algae extract (MAE)‐treated bone marrow‐derived dendritic cells (BMDCs) and bone marrow‐derived macrophages (BMDMs). BMDCs and BMDMs from BALB/c mice were pre‐treated with 0.1 μg/mL monophosphoryl lipid A (MPL) or 20 μg/mL of each MAE. After 2 days, the activated dendritic cells (a) and macrophages (b) were co‐cultured with CFSE‐labeled allogeneic naïve T‐cells, which were harvested from the spleen of C57BL/6 mice after 5 days. After co‐culture, the percentage of CFSE‐negative cells in either CD3^+^ CD4^+^ T‐cells or CD3^+^ CD8^+^ T‐cells was acquired by flow cytometry and analyzed by FlowJo, and cell supernatant was used to measure IFN‐γ secretion, using ELISA. The representative data obtained from the flow cytometry analysis were shown in histograms. Data are shown as mean ± standard error. One‐way ANOVA and Tukey's multiple comparison tests were performed for statistical analysis. *p < .0332; **p < .0021; ***p < .0002; and ****p < .0001 compared to control group.

To evaluate the functional activation of MAE‐treated DCs and macrophages, allogeneic MLR assay was performed. Allogeneic lymphocytes were co‐cultured with each MAE pre‐treated DCs or macrophages for 5 days, and the proliferation and INF‐γ production of T‐cells were measured. We observed that PY‐, SP‐, PC‐, MP‐, and CO‐treated DCs induced significant CD4+ and CD8^+^ T‐cell proliferation and PY‐, PC‐, and MP‐treated DCs enhanced IFN‐γ production of T‐cells (Figure 4a). However, CW pre‐treatment did not induce CD4^+^ T‐cell proliferation or IFN‐γ secretion. In macrophages, only PC treatment significantly induced both CD4^+^ and CD8^+^ T‐cell proliferations. PY‐ and MP‐treated macrophages promoted CD4^+^ T‐cell proliferation, but they did not promote CD8^+^ T‐cell proliferation. SP, CW, and CO treatments of macrophages did not induce T‐cell proliferation (Figure 4b). However, PY‐, PC‐, MP‐, and CO‐treated macrophages induced IFN‐γ production after co‐culture. Therefore, each MAE exhibited different activation capacities for DCs and macrophages, with PC treatment being the most effective in functional activation of both DCs and macrophages.

### 3.5 MAE injection promoted cytokine and chemokine production in peritoneal cavity

**FIGURE 5:**
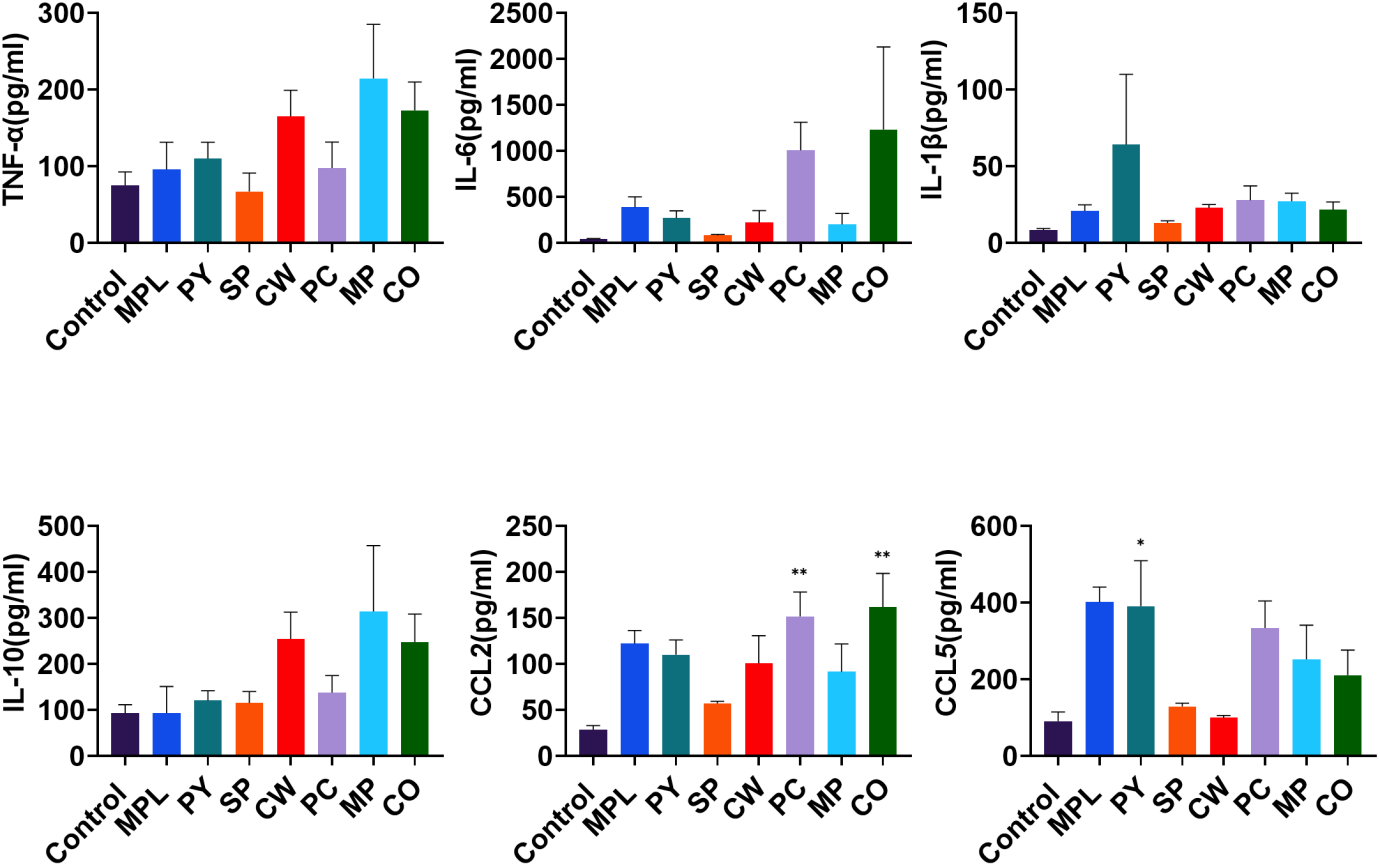
Cytokine and chemokine levels after intraperitoneal injection of marine algae extracts (MAEs) in mice. BALB/c mice (*n* = 6) were intraperitoneally injected with monophosphoryl lipid A (MPL) (1 μg/mouse) or MAEs (100 μg/mouse). The levels of each cytokine and chemokine in the supernatant of the peritoneal exudates and peritoneal lavage were detected by ELISA after 6 h. Data are shown as mean ± standard error. One‐way ANOVA and Tukey's multiple comparison tests were performed for statistical analysis. *p < .0332 and **p < .0021 compared to control group.

To examine in vivo immune stimulatory effects of MAEs, we injected MAEs (100 μg/mouse) intraperitoneally, and cytokine and chemokine levels in the peritoneal cavity were measured at 6 h post‐injection. MPL (1 μg/mouse) was used as a positive control as well (Figure 5). We observed that the levels of pro‐inflammatory cytokines TNF‐α, IL‐6, and IL‐1β had increased in the peritoneal cavity of MAE‐treated mice, but the difference was not significant. CW, MP, and CO treatments induced TNF‐α production; PC and CO treatments enhanced IL‐6 levels; and PY treatment elevated IL‐1β levels. Moreover, the anti‐inflammatory cytokine IL‐10 was produced in CW‐, MP‐, and CO‐injected mice. Chemokines, which were correlated with inflammatory cell recruitment, were also induced by PY, PC, MP, and CO treatments, but not in SP‐ and CW‐treated mice; only PY treatment provided statistically significant data (Figure 5).

### 3.6 MAEs induced innate immune cell recruitment in peritoneal cavity

**FIGURE 6:**
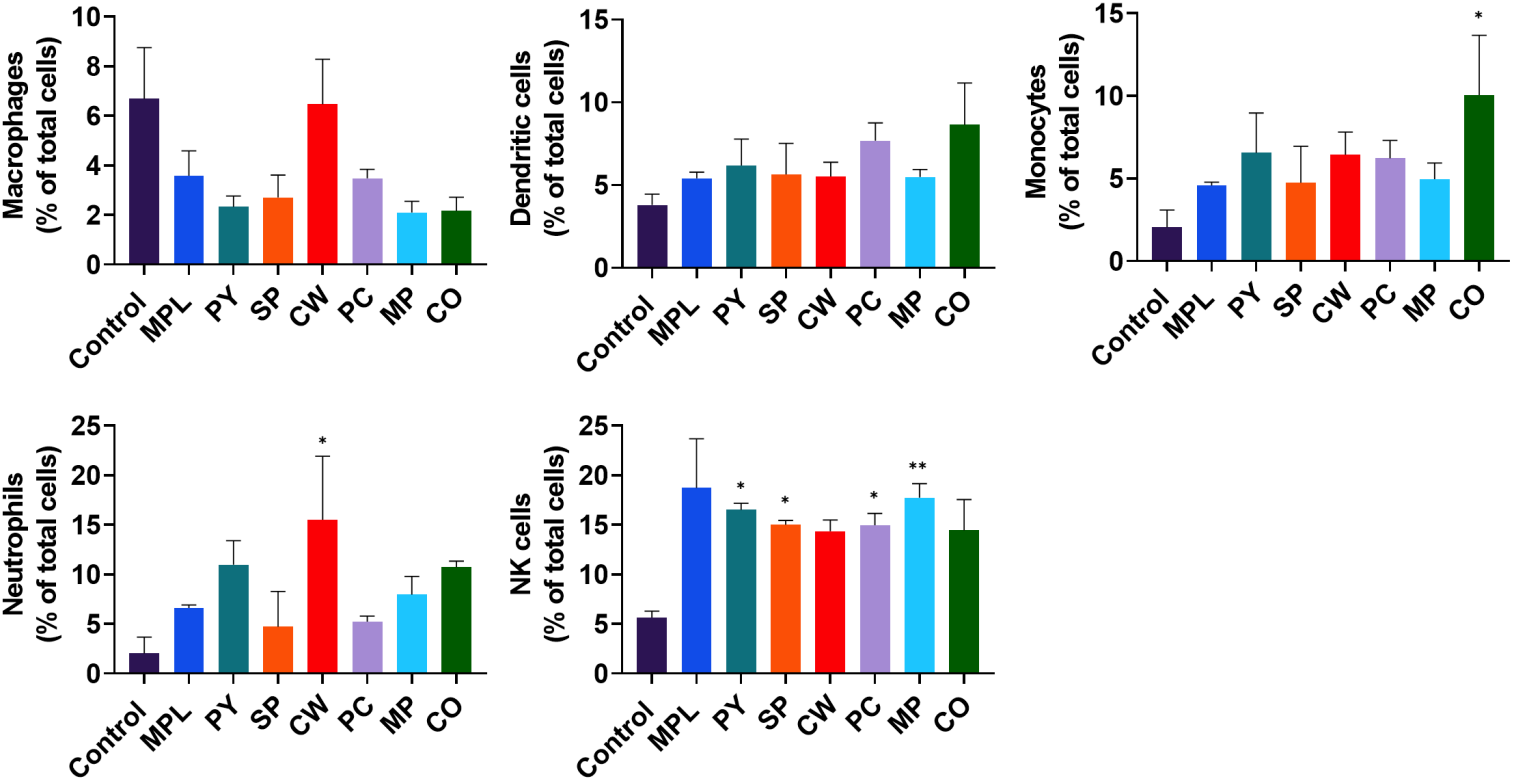
Cell population in the peritoneal cavity of mice after injection with marine algae extracts (MAEs). BALB/c mice (n = 6) were intraperitoneally injected with monophosphoryl lipid A (MPL) (1 μg/mouse) or MAEs (100 μg/mouse). Peritoneal cells were collected from peritoneal lavage after 24 h of MAE injection via centrifugation. The percentage of dendritic cells, macrophages, monocytes, neutrophils, and natural killer cells in the peritoneal cavity was measured by flow cytometry and analyzed by FlowJo. Data are shown as mean ± standard error. One‐way ANOVA and Tukey's multiple comparison tests were performed for statistical analysis. *p < .0332; **p < .0021 compared to control group.

We examined innate immune cell infiltration in the peritoneal cavity 24 h post MAE injection, using flow cytometry (Figure 6). In the control mice, the macrophages were found to be the highest cell population in the peritoneal cavity. The percentage of macrophages decreased when other immune cells were recruited to the peritoneal cavity following MAE treatment. All six MAEs recruited DCs, monocytes, neutrophils, and natural killer (NK) cells in the peritoneal cavity. CO treatment significantly increased the monocyte population; CW induced neutrophils; and PY, SP, PC, and MP treatments recruited NK cells in the peritoneal cavity. These data suggest that MAEs effectively stimulated immune responses and cell activation in vivo.

## 4 DISCUSSION

In this study, we investigated the immunoregulatory effects of six MAEs, specifically in promoting the activity of APCs. All six MAEs enhanced the expression of DC and macrophage activation markers and pro‐inflammatory cytokine production. Activated DCs and macrophages induced enhanced allogeneic T‐cell proliferation. In addition to the in vitro immunostimulatory effects on APCs, in vivo treatment with the six MAEs increased immune cell recruitment in the peritoneal cavity of mice.

According to the MTT assay, most MAEs in this study did not exhibit toxicity in BMDCs and BMDMs, but PC exhibited a slight decrease in the viability of both DCs and macrophages; however, we used 20 μg/mL as the treatment concentration for each MAE to ensure uniform conditions. Despite its cytotoxicity, PC treatment exhibited the highest in vitro APC activation and cytokine production, as well as in vivo cell recruitment. The total PC extract appeared to contain both cytotoxic and immunostimulatory components, and further research is required to separate the immunostimulatory components from the PC extract to reduce cytotoxicity. *Peyssonnelia* species contain peyssonoic acids, which have antimicrobial properties and inhibit the growth of human ovarian cancer cell lines (Lane et al., 2010). Moreover, this study highlights the pharmaceutical potential of PC as an immunostimulator. However, SP treatment, which exhibited toxicity against DCs and macrophages, could not induce immune cell activation both in vitro and in vivo, which was similar to that by other MAEs.

CW has been known to have high phenolic contents and exhibits antimicrobial activity against *Cutibacterium acnes* (Lee et al., 2021). In this study, its extract exhibited distinct effects on cell viability and immune cell activation. It had no cytotoxic effects, and increased viability of DCs and macrophages in a dose‐dependent manner (Figure 1). It induced the highest increase in activation marker expression on DCs (Figure 3a). However, the expression of activation markers on macrophages was not elevated by CW treatment (Figure 3b), and it induced low to moderate levels of cytokine production and T‐cell proliferation (Figures 2 and 4). This result suggests that CW can modulate signaling pathways related to cell viability rather than cell activation and function. Thus, further studies are required to investigate the pathways activated by CW.

PY is a well‐known red alga, which is a popular food source in Korea and Japan. PY contains carotenoids that are commensurate with the levels of flavonoids and phenolic compounds (Ha et al., 2020). Previous studies have reported the anti‐inflammatory, antitumor, and anti‐aging properties of PY (Ha et al., 2020; Lee et al., 2014; Lee, Kim, Choi, et al., 2015; Lee, Kim, & Nam, 2015; Park et al., 2015; Wang et al., 2020). Another red alga, MP, exhibits antimicrobial and B cell‐stimulating activities (Lee et al., 2013, 2021; Liu et al., 1997). Anthocyanin is one of the unique components of MP, in addition to a substantial concentration of phenolic compounds and flavonoids (Shin et al., 2006). In this study, both PY and MP induced moderate to high levels of activation marker expression, cytokine production, and T‐cell proliferation in APCs, suggesting the immunostimulatory potential of these algae.

*Caulerpa* species are widely used as soil fertilizers and food sources (Zubia et al., 2020). Moreover, several previous studies have reported their antioxidant activities (Li et al., 2012; Tanna et al., 2018). Recently, novel bioactivities of CO such as anti‐adipogenesis, anti‐inflammation, glucose metabolism regulation, and increase in insulin sensitivity have been reported (Manandhar et al., 2020; Sharma et al., 2017). In terms of APC activation, CO did not exhibit any significant results in our in vitro study; however, after the in vivo intraperitoneal injection of CO, high cytokine and chemokine production and the highest DC and monocyte recruitment in the peritoneal cavity of mice were observed. These data suggest that CO can create an environment in which immune cells can be activated, although it does not directly activate immune cells.

In this study, we evaluated six different MAEs for their in vitro immunostimulatory effects on APCs and in vivo immune cell recruitment. All six MAEs induced in vitro cytokine production, activation marker expression, and functional activation of APCs as well as in vivo immune cell infiltration in the peritoneal cavity. However, each extract exhibited distinct immunostimulatory patterns. This suggests that different underlying mechanisms and signaling pathways are involved with the activity of each MAE. This is the first study to determine and compare the immunomodulatory activities of six MAEs in both in vitro and in vivo systems. Further studies are required to elucidate the detailed mechanisms of action and the potential active ingredients of MAEs to broaden their biomedical applications. Our future research strategy includes conducting an in‐depth analysis of the effects and functions of each MAE. Moreover, we aim to study immunomodulatory capabilities of each individual component present within these extracts. Another key area of our investigation will focus on assessing their efficacy in supporting vaccines and their potential roles as novel adjuvant candidates in murine models.

## AUTHOR CONTRIBUTIONS

**Thi Len Ho:** Conceptualization (equal); data curation (equal); formal analysis (lead); investigation (lead); methodology (equal); visualization (lead); writing – original draft (lead); writing – review and editing (lead). **Jueun Lee:** Investigation (equal); writing – review and editing (equal). **So Yeon Ahn:** Investigation (equal); writing – review and editing (equal). **Dong‐Ha Lee:** Investigation (equal); writing – review and editing (equal). **Woo‐Jin Song:** Validation (equal); writing – review and editing (equal). **Inhae Kang:** Supervision (equal); validation (equal); writing – original draft (equal); writing – review and editing (equal). **Eun‐Ju Ko:** Conceptualization (equal); data curation (equal); formal analysis (equal); funding acquisition (lead); investigation (equal); project administration (lead); supervision (lead); validation (lead); writing – original draft (lead); writing – review and editing (lead).

## FUNDING INFORMATION

This work was supported by the National Research Foundation of Korea (NRF) grant funded by the Korean government (MSIT) (2020R1F1A1073040 and RS‐2023‐00211504) and Basic Science Research Program to Research Institute for Basic Sciences (RIBS) of Jeju National University through the NRF funded by the Ministry of Education (2019R1A6A1A10072987).

## CONFLICT OF INTEREST STATEMENT

The authors declare that there is no conflict of interest.

## Supporting information


Figure S1.

## Data Availability

The data that support the findings of this study are available from the corresponding author upon reasonable request.
