# Supplementary material for: Immunostimulatory effects of marine algae extracts on in vitro antigen‐presenting cell activation and in vivo immune cell recruitment
Source: Food Sci Nutr. 2023 Aug 21;11(10):6560–70. doi: 10.1002/fsn3.3605 (PMC10563723; doi:10.1002/fsn3.3605)
Supplement: Supplementary file 1 — Figure S1. [file FSN3-11-6560-s001.pdf]

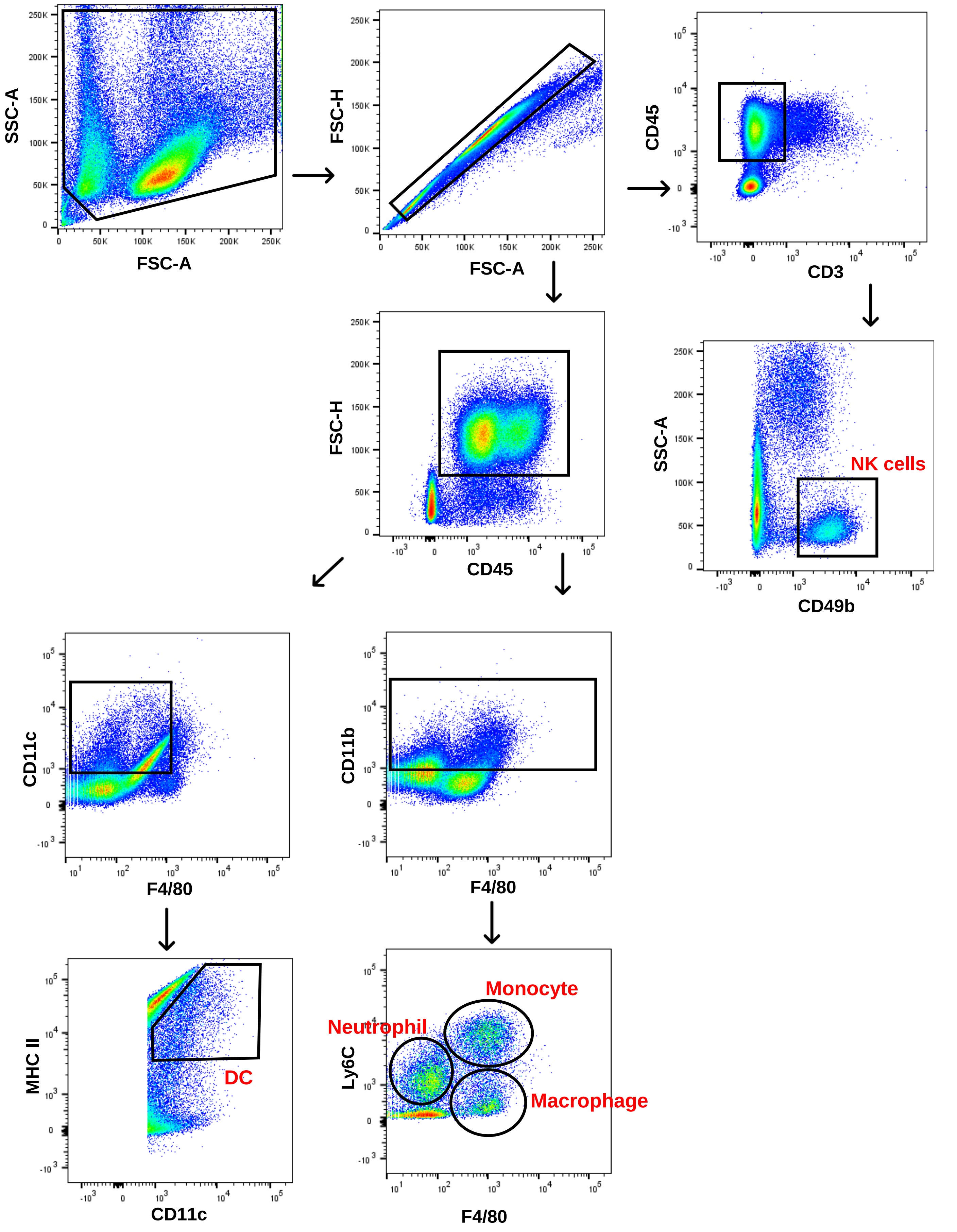

Supplementary Figure 1. Gating strategies for immune cells in peritoneal cavity. The harvested peritoneal cells after 24 hours of the MAE injection were stained with fluorescence-labeled antibodies and analyzed using flow cytometry. Macrophages; $CD45^{+}CD11b^{+}F4/80^{+}$, Monocytes; $CD45^{+}CD11b^{+}Ly6c^{high}$, Neutrophils; $CD45^{+}CD11b^{+}Ly6c^{int}$, DCs; $CD45^{+}CD11c^{+}F4/80^{-}MHCII^{+}$, NK cells; $CD45^{+}CD3^{-}CD49b^{+}$
